# Strengthening effectiveness evaluations to improve programs for women, children and adolescents

**DOI:** 10.1080/16549716.2021.2006423

**Published:** 2022-09-13

**Authors:** Agbessi Amouzou, Jennifer Bryce, Neff Walker, the JHU RADAR Team*

**Affiliations:** Department of International Health, Bloomberg School of Public Health, Johns Hopkins University, Baltimore, Maryland, USA

**Keywords:** Reproductive, maternal, newborn, child, adolescent health, nutrition, program evaluation, accountability, effectiveness evaluation

## Abstract

A full understanding of the pathways from efficacious interventions to population impact requires rigorous effectiveness evaluations conducted under realistic scale-up conditions at country level. In this paper, we introduce a deductive framework that underpins effectiveness evaluations. This framework forms the theoretical and conceptual basis for the ‘Real Accountability: Data Analysis for Results’ (RADAR) project, intended to address gaps in guidance and tools for the evaluation of projects being implemented at scale to reduce mortality among women and children. These gaps include needs for a framework to guide decisions about evaluations and practical measurement tools, as well as increased capacity in evaluation practice among donors and program planners at global, national and project levels. RADAR aimed to improve the evidence base for program and policy decisions in reproductive, maternal, newborn and child health and nutrition (RMNCH&N). We focus on five linked methodological steps – presented as core evaluation questions – for designing and implementing effectiveness evaluation of large-scale programs that support both the needs of program managers to improve their programs and the needs of donors to meet their accountability responsibilities. RADAR has operationalized each step with a tool to facilitate its application. We also describe cross-cutting methodological issues and broader contextual factors that affect the planning and implementation of such evaluations. We conclude with proposals for how the global RMNCH&N community can support rigorous program evaluations and make better use of the resulting evidence.

## Background

Evaluations of the effectiveness of programs being implemented at scale under real-world, non-trial conditions are essential to continue and expand recent gains in the health and nutrition of women and children, especially those living in low-resource or conflict settings. Such evaluations go beyond global public health metrics and produce information relevant to program improvement at the local and national levels [1]. Examples of learning that could only have been produced through well-planned, large-scale evaluations include the finding that the absence of a program component to promote community demand undermined the potential impact of the integrated Community Case Management (iCCM) strategy in Ethiopia [2], and that poor-quality training and health system supports vitiated a similar iCCM program effort in Burkina Faso [3].

Few large-scale effectiveness evaluations are conducted [4]. Too often, those with the power to plan and fund such evaluations assume that evidence of intervention effectiveness is sufficient to guarantee health and nutrition impact, and overlook critical implementation factors that determine whether proven interventions actually reach those who need them, at the levels of quality and intensity necessary to have a measurable impact on population health. Some have also suggested that advocates for a particular intervention or strategy may not support their evaluation because the results may weaken political or budgetary support [5].

Furthermore, large-scale RMNCH&N evaluations frequently fall short of the minimum technical standards needed to produce accurate and actionable conclusions, either at the level of the individual program or when data from multiple programs are concatenated to support higher-level estimates of impact [6]. A recent review of a random selection of impact evaluations commissioned or conducted by five major funders and published between 2009 and 2014 found that only about one-third met scientific standards for validity and reliability in sampling and analysis [4]. The authors also report that the great majority of these evaluations focused on intermediate process variables [such as service quality and coverage] rather than impact [4], which can lead to false conclusions about the extent to which a program is effective in improving population health.

Given the challenges and cost of conducting full RMNCH&N effectiveness evaluations, there is also an important and too-often-missed opportunity for learning from the many less ambitious assessment activities conducted or commissioned by individual donors, projects and programs. At present, there is no registry for RMNCH&N evaluations, and no systematic effort to compile results and use them to inform policies and programs. Work over the last decade by the multi-partner ‘Countdown to 2030 for Women’s, Children’s, and Adolescents’ Health’ [7] and others to define a limited set of RMNCH&N intervention coverage indicators that – if measured appropriately [8] – can produce valid information comparable across settings and over time is a useful starting point. For many RMNCH&N funders and implementers, however, decisions about how best to utilize the limited resources they have available for evaluation work are difficult, and advice from technical experts rarely takes into account budget limitations and non-technical factors that affect the design and funding of evaluation activities (e.g. the need for positive results within short time frames that can be used to demonstrate ‘success’) [9].

In this paper, we aim to encourage further commitment to and investment in high-quality effectiveness evaluations of RMNCH&N programs by providing a clear conceptual approach and summarizing the availability of new tools and methods to support its application. Many of these resources have been developed, tested and refined as part of the ‘Real Accountability: Data Analysis for Results’ (RADAR) project (Box 1), a five-year effort supported by Government Affairs Canada (GAC) to improve the evidence base for effective programming for women and children by developing and testing core metrics and new tools for RMNCH&N program accountability. We focus on five linked methodological steps – presented as core evaluation questions – for designing and implementing effectiveness evaluations of large-scale RMNCH&N programs that support program managers’ motivations for effective programming while ensuring transparent and rigorous accountability by donors. We also describe cross-cutting technical issues and broader, non-technical factors that affect when and how such evaluations are funded, designed, conducted and used to improve programs and policies affecting women and children.

**Box 1.** The RADAR project.

RADAR was designed in 2015 to address three specific technical gaps in the evaluation of programs for women and children in LMICs. The need for a clear and practical framework to guide decisions about evaluations: what core questions need to be addressed, which priority indicators should be measured, and what are ‘right-sized’ evaluation design options given time and resource limitations. The absence of simple, focused, practical tools specifically designed to generate sound evidence responding to the core evaluation questions. Major survey programs supported by global partners are time- and resource-intensive, and produce far more information than needed or able to be fully analyzed and reported by LMIC evaluation teams. Efforts by individual projects to generate *ad hoc* evaluation tools often – and understandably – do not produce results that are comparable across programs, settings or time, and sufficiently valid to support sound program and policy decisions. Insufficient capacity in evaluation thinking, design and implementation among donors and program planners at global, national and project levels.The RADAR project was designed in collaboration with Government Affairs Canada (GAC) to fill these gaps. The RADAR aim was to develop, apply and refine tools and approaches to increase the availability of accurate data in LMICs for evaluating country programs in reproductive, maternal, newborn and child health and nutrition (RMNCH&N) that can be ’rolled up’ to respond to GAC accountability needs.The RADAR team has developed a suite of compatible tools for use in large-scale evaluations in LMICs designed to produce answers to a set of core evaluation questions about RMNCH&N programs (Fig 1b). RADAR collaborated with partner organizations in Malawi, Mali, Mozambique and Tanzania (selected countries where Canada has RMNCH&N investments) to field test and refine the use of these tools in generating high-quality, complete, gender-sensitive, and relevant data. The RADAR team has developed a set of on-line Coursera classes covering the fundamentals of RMNCH&N program evaluation, and specific tools and methods for addressing the five core questions.More information on RADAR and access to RADAR tools is available at https://www.jhsph.edu/research/centers-and-institutes/institute-for-international-programs/current-projects/RADAR/.

## Answering five core evaluation questions about RMNCH&N programs

Program managers, governments and donors have overlapping information needs relative to RMNCH&N programs. In 2010, MNCH&N partner organizations agreed on a common framework to guide program evaluation (Figure 1a) [10]. In 2015, the same partners used this framework to agree on a sequential set of core evaluation questions (Figure 1b) and standard indicators [11]. The core questions are sequential and cumulative – like a staircase [12] – with a positive response to one question serving as a prerequisite for moving on to the next. Many donors now require their funded programs to define and measure specific processes, outputs and outcomes. Much rarer is the use of an explicit impact model with linked and sequential evaluation questions that generates evidence to explain why and how program activities result – or fail to result – in expected levels of health impact at population level.

**Figure 1a.**
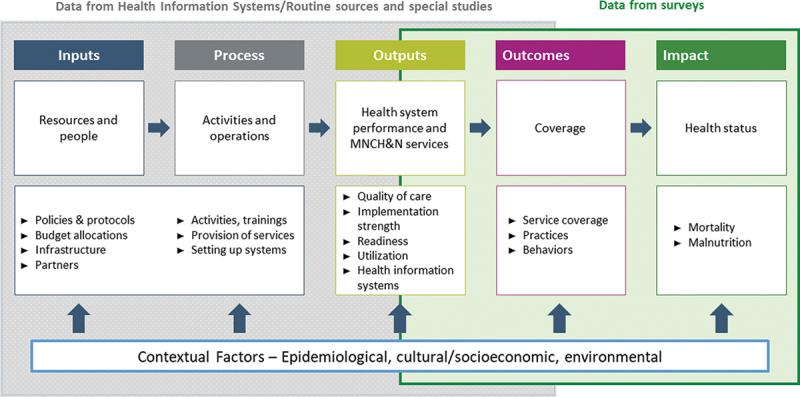
A common framework for evaluating the scale-up for maternal and child survival.

Source: Bryce et al., Evaluating the scale-up for maternal and child survival: a common framework. *International Health* 2011: **3**(39–146).

**Figure 1b.**
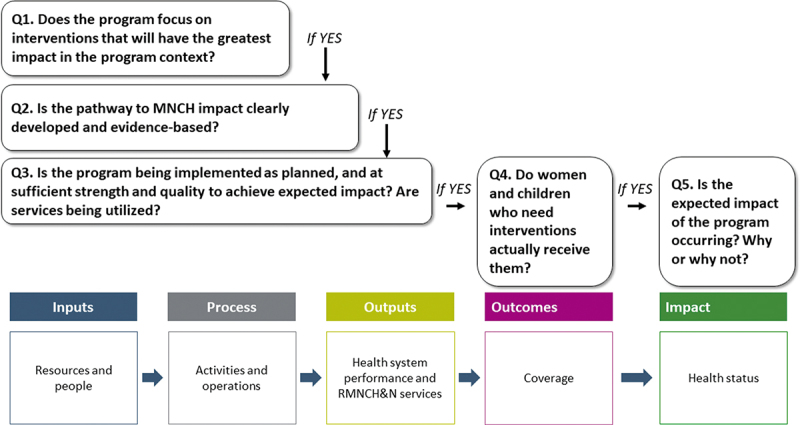
Core questions about RMNCH&N programs for program managers, governments and donors, related to the common framework for evaluating the scale-up for maternal and child survival.

RADAR provides specific guidance and tools to help program managers in LMICs and their donors respond to the five core evaluation questions efficiently, with reporting broken out by key equity variables (e.g. sex, geographic region, household economic status). RADAR focused first on helping country programs collect, analyze and use data relative to these questions so that they could improve their programs while promoting the use of indicators and methodologies amenable to rolling-up results to meet Canada’s commitments relative to the broader RMNCH&N accountability agenda [13].

### Question 1: does the program focus on interventions that will have the greatest impact in the program context?

RMNCH&N programs must use their limited resources to deliver interventions that match the specific health and nutrition needs of women and children in their context. Although this may seem obvious, it is often not the case. Examples include programs in low child-mortality settings that focus on improved breastfeeding as a way to reduce deaths among under children under five [14], or programs in settings where large proportions of under-five deaths occur in neonates that do not include interventions targeting very young infants [15]. The first step in any large-scale effectiveness evaluation is to assess whether the interventions delivered through the program address the major causes of morbidity and mortality among women and children. If yes, evaluators can move forward to the next core question. If no, the program will not result in health impact at population level, and this should be discussed with stakeholders. There may be situations in which health conditions or risks may be priorities for program targeting, even though they might not be a major cause of morbidity and mortality for most women and children (e.g. obstetric fistula). The Lives Saved Tool (*LiST*) is purposively designed to support this type of analysis and the design of programs that will save women’s and children’s lives. LiST provides users with up-to-date estimates of the effectiveness of RMNCH&N interventions on specific causes of and risk factors for maternal and child deaths, as well as epidemiological profiles and estimates of intervention coverage at national and – where available – subnational levels in LMICs [16].

### Question 2. Is the pathway to RMNCH&N impact clearly developed and evidence-based?

RMNCH&N programs being delivered at scale in low-resource countries often fail to produce measurable impact. One reason for this failure is that neither the program nor the evaluation team have committed to a fully specified program impact pathway, describing the range of activities and steps that must be completed successfully for the potential impact of the focus interventions to realize their expected effects on health and nutrition. We cited two examples of incomplete impact models resulting in impact failure above [2,3]. The RADAR project includes two tools to help policy-makers and practitioners develop fully specified impact models for their RMNCH&N programs without ‘reinventing the wheel’ for each program cycle. The RADAR *Evaluation Planning Tool* allows users to create, edit and share impact models [17], and the RADAR *LiST Visualizers* allow users to explore examples of previously developed impact models, overlaid with relevant annotations [18]. Fully specified and evidence-based impact models are a prerequisite for valid conclusions about the effectiveness of RMNCH&N interventions and strategies.

### Question 3. Is the program being implemented as planned, and at sufficient strength and quality to achieve expected impact? Are services being utilized?

Several large-scale effectiveness evaluations conducted over the past decade have demonstrated the importance of assessing each of the separate elements of the program impact model – implementation strength, service quality and service utilization – and combining the results to produce an estimate of expected impact [2,3,19–21]. Returning to the concept of evaluations built on a staircase of core questions introduced above and shown in Figure 1b, a well-designed program cannot be expected to produce impact if it is not implemented as planned (sometimes referred to as ‘fidelity’ [22]), is feasible for full implementation in the program setting and is acceptable to the target population as reflected in measures of satisfaction with services. Even if implemented well, a program cannot produce impact if the quality of intervention delivery is inadequate to ensure biological or behavioral effectiveness. And even if implemented well and at high quality, interventions and strategies cannot produce improvements in population health if the services are not utilized by the target population. Measurements or estimates of all three impact model elements – implementation strength, quality, and utilization – are therefore essential in any full evaluation of program impact. The availability of tools for assessing these elements has expanded rapidly in recent years [23], and increasing numbers of evaluations that demonstrate how the use of a suite of integrated tools to assess elements of a program impact model can lead to richer, ‘thicker’ [24] information to guide program improvement [25,26]. The availability of new RADAR tools will contribute to ensuring that these essential elements are included in future RMNCH&N program evaluations [27].

### Question 4. Do women and children who need interventions actually receive them?

Coverage, defined as the proportion of the population in need of a health intervention who receives it, is an essential requirement for impact and must be included in the plan for any evaluation of RMNCH&N program impact. By definition, coverage must be measured at the population level, and therefore requires population-based data most often obtained from household surveys. Recent advances in the methodological issues surrounding coverage measurement have led to a better understanding of the limitations of available measurement methods, and the refinement of globally recommended standard indicators for RMNCH&N intervention coverage [7]. Existing survey programs such as the Multiple Indicator Cluster Survey (MICS) [28] or the Demographic and Health Survey (DHS) [29] are an important resource for many countries and partners but are not always available for the time frames or specific sub-populations needed in a program evaluation. The MICS and DHS tools are also designed to measure a large number of indicators that go far beyond what is needed for most RMNCH&N programs, and are too expensive for most governments and implementing agencies to conduct independently. The RADAR team has therefore developed a coverage survey tool that can be adapted to produce program-specific coverage measurements consistent with RMNCH&N indicators measured through MICS and DHS, but that is tightly focused on a subset of priority RMNCH & N indicators able to be measured accurately through household surveys [30]. The RADAR coverage survey tool is also modular, permitting selection of subsets of questions that reflect the intervention areas addressed by specific programs.

Piloting of the RADAR coverage tool by partners highlighted the many technical and logistical challenges confronting program implementers who decide to conduct a survey. The tool therefore includes a set of resources to facilitate survey planning, implementation, and analysis (https://www.radar-project.org/coverage-survey).

The science of coverage measurement has advanced over the past 5 years since RADAR was designed. It is now clear that accurate population coverage estimates must be linked to earlier elements in the RMNCH&N impact model – service quality, service utilization, and client adherence to recommendations – if they are to serve as valid predictors of health and nutrition impact. A recent review highlights the advantages and constraints of moving from crude coverage to quality-, utilization- and adherence-adjusted coverage measurements, calling for increased standardization of indicators and terminology, and improved measurement methods [19]. This approach is entirely consistent with the common framework, five core questions and tools presented here, and represents an important step forward in promoting the inclusion of measurements (or estimates) of all elements in a program impact model when conducting an evaluation.

### Question 5. Is the expected impact of the program occurring? Why or why not?

The final core evaluation question often, and appropriately, receives the most attention. Did the program lead to population-level impact, defined as measurable and significant changes in the health and nutrition of women and children? Those who have funded multi-site, multi-method, program impact evaluations in the past now appear to be focusing their efforts on earlier steps in the program impact model, perhaps to avoid the potential conflicts and costs associated with impact assessments. An assessment of the quality of recent evaluations supported or commissioned by aid agencies found that over 90% focused on program performance, with the remainder divided between those that focused on impact (7%) or both performance and impact (2%) [1].

For programs within which the previous four core evaluation questions have been answered affirmatively, impact can be expected – if sufficient time has elapsed to allow the program to result in measurable changes at the population level. But can this impact be measured reasonably well and at reasonable cost by individual programs and their partners? Many of the health events that are of greatest importance for women and children – deaths, early births, and changes in nutritional status – are relatively rare events, requiring enormous sample sizes and resource-intensive measurement strategies that extend over long periods and are not feasible for use in most program evaluations. In settings where MICS, DHS, or other ambitious measurement efforts have been carried out, program evaluators can often access the data to conduct follow-up analyses that produce measured impact results relevant to their specific program. In most evaluations, however, this is not possible, and the expected impact can only be confirmed through modeling. The *LiST* tool was originally designed to meet this need [31], and has become a popular tool for modeling the impact of RMNCH&N interventions. The RADAR team has developed an on-line version of the *LiST* tool with greatly expanded accessibility and user support [10].

Although LiST OnLine may not be the sole solution, a clear focus on health and nutrition impact is essential for all RMNCH&N evaluations. Even if time or resources preclude impact measurement, alternatives now exist. LiST and other modeling tools, and firm adherence to the use of an impact model that can support plausible inferences about program impact using available data [32], can provide scientifically defensible estimates of population-level impact. Program data alone cannot meet this need, as they reflect only a portion of the target population who are in contact with the program, but can be useful if placed in the context of a fully defined impact model accompanied by explicit statements of their limitations.

## Cross-cutting technical issues specific to evaluating a program at scale

Despite their public health importance, rigorous evaluations of RMNCH&N programs being implemented at scale in low-resource settings are rare, and little attention has been directed at defining and addressing the special methodological challenges they present over and above evaluations conducted under more controlled conditions. Table 1 identifies some of the most important differences between large-scale evaluations and randomized- or cluster-randomized trials, and summarizes the implications of each for evaluation design and implementation. We have categorized these challenges here as related to design, timing, sample size and documentation. Although some of these challenges arise most often in the context of evaluations conducted by groups external to the implementing agency, they are also important as considerations for internal evaluations, even those focused on single impact model elements such as implementation strength. RADAR seeks to improve the quality of evaluations conducted by all groups, including relatively small implementing organizations planning relatively simple, ‘adequacy’ evaluations [32].

**Table 1.** Methodological challenges of evaluations of RMNCH&N programs being implemented at scale.

Characteristic of large-scale evaluations	Implications for evaluation design and implementation
Evaluators rarely control the location, timing, and strength of program implementation.	Limits use of randomized designs or designs that require a planned schedule for program implementation (e.g. randomized stepped-wedge designs). Definition of true comparison groups is often difficult and sometimes impossible. Reinforces the importance of measuring the strength of implementation and quality of services. In prospective evaluations, early evidence of inadequate implementation in a stepwise design may call into question the need to conduct assessments of later impact model elements, including impact. Schedule for evaluation activities must be sufficiently flexible to adapt to unforeseen changes in the program calendar.
Most programs consist of several interventions implemented simultaneously.	Evaluating multiple individual interventions within a program may require prohibitively large sample sizes. Different interventions within a program may require more or less time to achieve expected results, and may be synergistic or antagonistic relative to expected outcomes or impact.
The pathways from activities to outcomes and impact are often long and complex.	Adequacy evaluations [31] to determine whether expected process and outcomes were met are essential and contribute to learning but are rarely sufficient to support inferences about attribution or even contribution. Most evaluations will require multiple sources of data or information that must be coordinated over time and assessed for quality and relevance.
Feedback from evaluation results may lead to changes in the intervention over time.	Although program improvement as a result of interim evaluation findings is desirable, changing the intervention or strategy during the evaluation period is an important threat to both the internal and external validity of the findings. Careful, continuous documentation of program activities and timing, evaluation implementation and contextual factors is required to counter these threats to validity, and requires resources and assiduous attention.
Contextual factors may play a more important role than in more controlled evaluations.	Contextual factors must be included in the evaluation plan and design from the outset – whether documented using existing data sources or, if resources permit, measured as an integral part of the evaluation. Sample sizes may need to be enlarged to take into account the need to stratify by relevant contextual factors.

***Design*** challenges in conducting large-scale RMNCH&N evaluations reflect those in the broader sphere of causal inference in public health [e.g. 32–34], including the difficulties involved in defining true counterfactuals or comparison groups and maintaining them over time given the evolution of the program and context – sometimes in response to intermediate findings from the evaluation itself. Related challenges include meeting minimum assumptions for use of randomized designs or designs that require a predictable schedule for introduction of an intervention (e.g. stepped-wedge designs), and the fact that most ‘real-world’ programs have complex pathways to impact involving simultaneous implementation of more than one intervention. This demands not only multiple, coordinated data collection activities along the impact model, but also careful documentation of the program, evaluation activities and contextual factors. In prospective evaluations, another important challenge is that results from assessments of program design, implementation, quality, utilization or coverage may indicate that there is no reason to expect population-level impact to occur. In such cases, the resources required to measure or model impact might usefully be redirected to investigate reasons for the failure to achieve earlier steps in the impact model. Groups that opt for an ‘adequacy design’ with no comparison group should be aware of the limits this design places on the inferences able to be drawn from their results [31]. If time or resource constraints preclude a full effectiveness evaluation addressing all five core questions, program managers and donors should opt for answering questions about program implementation and quality *well*, and interpreting their findings in the context of a clearly specified impact model. Investing in less-than-rigorous measurement activities does not contribute and should be avoided.

***Timing*** is a critical challenge in large-scale evaluations for many reasons. For example, evaluators do not always control the launch or speed of intervention roll-out. Regardless of evaluator control over implementation calendars, unforeseen changes in program implementation may require quick changes in evaluation design, and interventions within a program can vary widely in the time required to result in measurable impact (as an example, consider the time needed for impregnated nets to reduce malaria mortality *vs*. the time needed for breastfeeding promotion interventions to yield improvements in nutritional status). Evaluating a program before the component interventions have a reasonable probability of achieving population impact is one of the most common mistakes in the effectiveness evaluations [35].

***Sample size*** is often a limiting factor in determining which of the core evaluation questions can be addressed, with what level of confidence, and whether changes in health outcomes or impact can be attributed to a specific intervention or strategy. Decisions about design – whether the feasibility of geographic or historical comparison areas, acceptable levels of precision and power, or measuring the impact of individual interventions *vs*. packages of interventions *vs*. delivery strategies for multiple interventions – all depend on the feasibility and affordability of obtaining adequate sample sizes for analysis. Sample size considerations often include the need to capture measurable changes in several interventions simultaneously, some of which may reflect relatively rare events or smaller target populations (e.g. exclusive breastfeeding among children under 6 months of age; neonatal and under-five mortality). Too often overlooked is the fact that stratification to support equity analyses or examine the effects of contextual factors requires enlarged sample sizes because each categorical subgroup requires adequate numbers to support meaningful conclusions.

***Documentation*** of program implementation, as well as contextual factors affecting intervention roll-out and potential effectiveness (e.g. socio-economic, geographic, environmental, and demographic features; health care infrastructure; other programs or activities that may affect RMNCH&N; other relevant events that may affect the health of women and children such as disasters, famines, migration, conflict, etc.) is needed to support attribution of positive outcomes to a program, and to rule out alternative explanations (internal validity), as well as to assess the extent to which the evaluation results might be generalizable to other settings (external validity) [36]. Documentation of evaluation activities is also needed to support comparisons with findings from other settings and programs and, ideally, to support replication of the evaluation findings. Too often, the tedious and resource-intensive work of documenting programs, evaluation activities and contextual factors is not explicitly included in evaluation plans and budgets, and therefore either does not happen or is insufficiently rigorous. This needs to change, and existing resources for strengthening documentation in large-scale evaluation must be given greater attention in teaching about, contracting for, and critically assessing the results of evaluations [37]. Evaluators must include documentation of both the program and evaluation activities as a specific, cost element in their plans.

An additional crucial technical issue is recognizing that even the best-designed evaluations, based on rigorous data related to the five core evaluation questions, is unlikely to provide a full explanation of why a specific strategy, program or intervention results in a particular level of population health impact. Additional complementary studies, using qualitative methods and political-historical-structural analysis, can enhance the understanding of ‘what works’ in RMNCH&N programs and provide important guidance for those seeking to improve program impact [38].

Despite these design challenges and costs, investments in rigorous evaluations of RMNCH&N programs being implemented in real-world contexts are essential, and provide good value in terms of informing future programs that will save maternal and child lives.

## The broader context for effective RMNCH&N evaluations at scale

Large-scale evaluations are affected not only by technical concerns such as those described above but also by characteristics of the broader context that affect which of the many potential evaluation questions are given priority, whether evaluations are adequately funded and by whom, and the ways in which large-scale evaluations are currently tracked. We highlight a selection of these contextual factors in this section.

***Funding***: One fundamental determinant of whether and how large-scale evaluations are conducted is the availability of sufficient funding. Rigorous, large-scale effectiveness evaluations of RMNCH&N programs *in situ* require multiple data collection activities over several years. They are expensive, challenging to design and implement, and require resource-intensive quality control. The resulting data sets can be fully utilized to generate learning only through advanced analytical techniques, and often produce mixed results – pointing to some successes, but in most cases also highlighting deficiencies in program design or implementation. For agencies or organizations that need fast and positive results to maintain their political or financial support, there are disincentives associated with this type of evaluation. They are risky, because they may produce results pointing to weaknesses in the program and its impact, jeopardizing continued funding [5]. These evaluations are also often long – extending over five to 10 years – because they must allow time for full implementation of interventions and for intervention effects to be translated into measurable changes in population health [39].

***The mirage of global public health metrics***: Inadequate funding for large-scale effectiveness evaluations is exacerbated by the incentive structures guiding such investments. *BMJ Global Health* has fostered a recent and welcome discussion of the tensions between the needs of aid agencies and governments to demonstrate success and the scientific standards that should guide the design and conduct of objective global health evaluations [6,40]. Potential supporters of in-depth effectiveness evaluations are also increasingly pressured to buy into the highly centralized global metrics enterprise, which generates high-level estimates of disease burden, but provides little or no information useful to program managers and governments about how best to meet the needs of their populations [1]. The value of full-scale evaluations of programs being implemented at scale rests in the opportunities they provide to learn from country-specific barriers to reaching women and children with interventions that can improve their health and development, and how these barriers can be overcome in specific contexts. Global public health metrics do not generate this learning and must be complemented by rigorous locally- and nationally focused program evaluations.

***Lack of a systematic tracking mechanism***: Current practices surrounding large-scale RMNCH&N evaluations are not organized in ways that promote the use of results to improve programs. There is no systematic registry for planned RMNCH&N evaluations, no systematic archive for results generated from these evaluations, and no systematic mechanism for synthesizing these results and making them available to those who can use them to improve policies and programs. Largely as a result, few current evaluations address the sustainability of programs [41] – something we have not addressed within the RADAR project.

We need a new, systematic approach to tracking large-scale effectiveness evaluations, driven by the need to strengthen the evidence base on how to deliver and sustain effective programs for women and children. Rather than relying on individual donors and their immediate priorities, RMNCH&N program effectiveness evaluations should be recognized as a global common good – adequately funded and based on an evolving, theory-driven framework that supports meta-analyses – with results communicated proactively to those making decisions about RMNCH&N policies, programs and investment strategies. This new approach should include three distinct and complementary components:

1. ***Registration of evaluation protocols***. A systematic tracking mechanism or registry for RMNCH&N evaluations, similar to that for clinical trials [42], is needed to document the objectives and designs of evaluation activities and avoid duplication.
2. ***Results repository for program evaluations***. The results of most program evaluations, and particularly internal evaluations conducted by implementing organizations, do not result in published, peer-reviewed publications, and are therefore not available to those who most need to learn from them. A repository, or archive, based on quality standards and perhaps even anonymized data sets, is needed as a foundation for learning.
3. ***Periodic, subject-specific knowledge syntheses*** gained from the evaluations. Ensuring data sets from large-scale evaluations are publically available would lay a foundation for syntheses of what is being learned. Cochrane provides this essential service for randomized controlled trials [43]. The Campbell Collaboration performs a similar service for a broader range of social science research [44], including some studies labeled as ‘evaluations’.

A set of targeted, multi-country, prospective, large-scale effectiveness evaluations could serve as the backbone of this initiative, with systematic incorporation of findings from more-targeted evaluations. The initiative would need to be led by an organization with deep, wide and multidisciplinary technical expertise, capable of maintaining independence from those who fund, implement and advocate for specific interventions, delivery strategies or programs. It would differ from past initiatives on behalf of women and children that combined political and technical roles, such as the Partnership for Maternal, Newborn and Child Health [45] or the more narrowly focused and time-limited Commission on Information and Accountability for Women’s and Children’s Health [46]. Better models – worth investigating – are the Clinical Trials Registry [42], the Cochrane Collaboration [43] or the Campbell Collaboration [44], in which the missions are more purely technical and the profiles of the managing organizations and funders are subservient to the public scientific service provided. Indeed, the Cochrane motto of ‘*Trusted evidence. Informed decisions. Better health*.’ would be an excellent starting point, as would the principles established by the Campbell Collaboration in 2017 [47].

## Conclusions

Rigorous impact evaluations of programs intended to improve the health and nutrition of women and children under real-world, non-trial conditions, produce essential information that is not available from other sources that is needed to inform program and policy decisions. The learning generated by these evaluations should be viewed as a ‘global common good’ to promote the health and nutrition of women and children. Ensuring accountability among aid agencies is also important, and we can do a better job of assisting these agencies in planning more focused, time-limited evaluation activities that are part of a common evaluation framework for RMNCH&N. RADAR has contributed by providing practical guidelines and tools to support these evaluations. Good evaluation data can serve as an important counterbalance to the growing dominance of global public health metrics with limited local and national relevance.

We need re-invigorated efforts to ensure agreement on a common evaluation framework for RMNCH&N, including evidence-based impact models and standard indicators that can be measured accurately using rigorous methods. Modeling tools that use the best available evidence on intervention effectiveness and local epidemiology, and can translate program gains in coverage and quality into estimates of impact need continued support. We also need a mechanism to ensure that the results from all RMNCH&N evaluations – both large and small – are registered and assessed for quality, made publicly available, and synthesized regularly to increase the knowledge base on how best to improve the health and nutrition of women and children.

## Paper context

There is wide recognition that rigorous evaluations of programs being taken to scale in low- and middle-income countries are needed to inform program and policy decisions. New in this paper is the use of a common evaluation framework with five core evaluation questions to guide evaluation planning and implementation, leading to results that are comparable across settings and over time and a set of methodological tools to help answer these questions.
